# A SPOTLIGHT ON ADAPTATION: PREIMPLEMENTATION OF MONTESSORI-BASED APPROACHES IN VA LONG-TERM CARE

**DOI:** 10.1093/geroni/igad104.1142

**Published:** 2023-12-21

**Authors:** Caroline Madrigal, Whitney Mills, Camilla Pimentel, Christine Hartmann, A Lynn Snow, Cameron Camp, Michelle Hilgeman

**Affiliations:** VA Boston Healthcare System, Boston, Rhode Island, United States; VA Providence Healthcare System, Providence, Rhode Island, United States; VA Bedford Healthcare System, Bedford, Massachusetts, United States; VA Bedford Healthcare System, Bedford, Massachusetts, United States; Tuscaloosa VA Medical Center, Tuscaloosa, Alabama, United States; Center for Applied Research in Dementia, Solon, Ohio, United States; Tuscaloosa VA Medical Center, Tuscaloosa, Alabama, United States

## Abstract

Effectively adapting evidence-based interventions for nursing home (NH) implementation is a critical, yet under-examined, component of improving care quality. Montessori-based activity programming (MAP) is an evidence-based intervention that promotes person-centered care, engages persons living with dementia, and mitigates distress behaviors. Currently, there is sparse examination of MAP in Department of Veterans Affairs NHs (i.e., Community Living Centers, CLCs). We report on the use of the Framework for Reporting Adaptations and Modifications-Enhanced (FRAME) to track adaptations made to MAP – providing a pre-implementation exemplar for NH clinicians and implementation scientists. Qualitative and quantitative data were collected across two phases (i.e., pre-implementation and pilot implementation) at eight VA CLCs between 2017-2019. We used an iterative, rapid content analytic approach to triangulate findings across data sources (e.g., advisory panel, staff interviews, training evaluations, field notes, fidelity assessments) and identify needed adaptations for the CLC setting and population. More than 300 frontline VA CLC staff participated in qualitative interviews and/or provided feedback on quantitative staff training. Thirty-six adaptations were made. Most adaptions occurred during the pre-implementation phase, were reactive, focused on training/evaluation, and involved researchers, intervention developers, and practitioners. All were fidelity-consistent with MAP. The most common goal across adaptations was increased reach/engagement of the intervention. CLCs and community NHs can use findings to support intervention adaptation, and adapt and implement MAP to improve meaningful engagement for persons living with dementia and other residents. Future research should further evaluate and standardize FRAME for diverse users of complex interventions.

